# Quantifying stent‐induced dose perturbations in intravascular brachytherapy using 3D‐ printed phantoms and film dosimetry

**DOI:** 10.1002/acm2.70432

**Published:** 2026-01-05

**Authors:** Jessica S. Jung, Lyu Huang, Nicholas Coupera, Yijian Cao, Jenghwa Chang

**Affiliations:** ^1^ Department of Physics and Astronomy Hofstra University Hempstead New York USA; ^2^ Northwell New Hyde Park New York USA; ^3^ Department of Radiation Medicine Northwell Health Lake Success New York USA; ^4^ Donald and Barbara Zucker School of Medicine at Hofstra/Northwell Hempstead New York USA

**Keywords:** 3D‐printed phantom, film dosimetry, intravascular brachytherapy

## Abstract

**Background:**

Coronary artery disease (CAD), the leading cause of death worldwide, is the narrowing of coronary arteries due to atherosclerotic plaque buildup. A common treatment for CAD is percutaneous coronary intervention (PCI), often involving stent placement. However, a common complication or in‐stent restenosis (ISR) can occur in 10%–20% of patients which call for the use of therapies like intravascular brachytherapy (IVBT). IVBT delivers targeted beta radiation, typically from Sr‐90/Y‐90 sources, to inhibit neointimal hyperplasia and reduce restenosis rates. Accurate dose delivery is critical to treatment success, but challenges such as source positioning and dose uniformity persist. Recent advances in 3D printing and radiochromic film dosimetry offer promising tools for more precise dose verification in IVBT, enabling high‐resolution assessment of dose distributions and stent‐induced perturbations.

**Purpose:**

IVBT requires precise position to ensure effective treatment. However, stents introduce complexities in dose distribution due to their material and geometry, which can lead to attenuation and impact treatment outcomes. This study aimed to quantify stent‐induced dose perturbations using a custom 3D‐printed stent phantom and Gafchromic EBT‐4 Film, providing insights for dosimetry of IVBT.

**Methods:**

Dose measurements were conducted using a custom designed 3D‐printed stent phantom. The film calibration was performed using the RIT film dosimetry package from 0 to 12 Gy. The phantom was designed for a Synergy XD Stent with a diameter of 3 mm with the Sr‐90/Y‐90 source catheter position designed to be in the center of the stent. Percent depth dose (PDD) distributions were modeled using the third‐order exponential polynomial function and compared with Monte Carlo simulations to evaluate agreement. Discrepancies were quantified using root mean square error (RMSE) and mean absolute error (MAE). The stent effect on PDD was analyzed using a paired *t*‐test, and a dose reduction factor (DRF) was calculated to assess attenuation.

**Results:**

The third‐order exponential polynomial function demonstrated an excellent fit for both configurations, with R‐squared values of 0.999 (no stent) and 0.999 (with stent). RMSE and MAE values were slightly higher for the with‐stent dataset (0.038 and 0.036, respectively), reflecting increased discrepancies. The paired *t*‐test showed a statistically significant difference between PDD values (*t* = −6.591, *p* < 0.0001). The average PDD difference between configurations was 4.26% in the clinically relevant region (2–5 mm). The DRF ranged from 1.18% to 7.92%, with an average attenuation of 4.5%.

**Conclusion:**

The presence of a stent significantly impacts dose delivery in IVBT, attenuating approximately 4.5% of the dose within clinically relevant depths. These findings highlight the importance of accounting for stent‐induced attenuation in treatment planning to ensure accurate dose delivery. The custom stent phantom demonstrates its usefulness in capturing dose perturbations, offering an effective tool for improving IVBT dosimetry.

## 1 INTRODUCTION

Coronary artery disease (CAD) results from the narrowing or blockage of coronary arteries due to the accumulation of atherosclerotic plaque—a complex mixture of cholesterol, fatty substances, cellular waste, calcium, and fibrin.^1^ This process often begins with endothelial damage caused by risk factors such as smoking, high cholesterol, high blood pressure, diabetes, or inflammation.^2^, ^3^ As plaque accumulates, it restricts blood flow and reduces oxygen delivery to the myocardium, potentially leading to ischemic events like angina or myocardial infarction. CAD remains the leading cause of death globally, accounting for approximately 610,000 deaths annually in the United States alone.^4^, ^5^ In 2022, the global prevalence of CAD was estimated at 315 million, and the mean annual per capita direct health care cost of CAD alone was 13,000 ± 730 2022 US dollars in the United States.^6^, ^7^ The projected costs are expected to triple between 2020 and 2050.^7^

**FIGURE 1:**
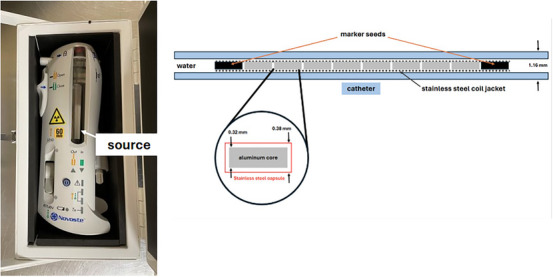
Dimensions of a single seed source and an 8‐seed train source assembly (right), shown alongside the corresponding Novoste Beta‐Cath 3.5F delivery system (left) (Best Vascular, Inc., Norcross, GA).

Intravascular brachytherapy (IVBT) using a delivery system that is illustrated in Figure 1 is an effective intervention for managing in‐stent restenosis (ISR), a recurrent complication following percutaneous coronary intervention (PCI) for treatment of CAD.^8^ ISR, defined as > 50% stenosis of a preciously stented segment, results from neointimal hyperplasia or excessive vascular smooth muscle cell proliferation (scarring) in response to arterial injury from stent placement or balloon angioplasty.^9^ Approximately 10%–20% of patients after PCI develop ISR.^10^ Despite advances in drug‐eluting stents, which decreased ISR rates, refractory restenosis cases still necessitate adjunctive therapies like IVBT.^11^

IVBT mitigates ISR by delivering localized ionizing radiation via catheter‐based systems, such as the Novoste Beta‐Cath system, which employs the Strontium‐90/Yttrium‐90 (Sr‐90/Y‐90) sources emitting beta particles with energies up to 2.27 MeV.^8^, ^12^ The radiation dose, typically 18.4 to 23 Gy, is prescribed based on the vessel diameter, with dwell times of 200 to 300 seconds.^13^ Studies have demonstrated that IVBT reduces restenosis rates from approximately 35%‐40% to below 10%,^14^ improving vascular patency and reducing the need for repeat revascularization. The outcome of IVBT highly depends on the precise delivery of the prescribed dose to the target area. Precise dosimetry is critical, as inadequate doses may result in treatment failure, while excessive exposure increases the risk of complications such as aneurysms or thrombosis.^15^ Previous studies have highlighted the challenges associated with ensuring dose uniformity along the source train, especially as the source length increases. A uniformity tolerance of ± 10% for doses delivered along two‐thirds of the treatment length is reported.^16^ Deviations in source positioning due to gravity or catheter tilting may also cause dose variations.

Radiochromic film dosimetry has proven effective in characterizing 2D dose distribution at a high resolution, particularly for high‐gradient, short‐range beta sources.^17^ Soares et al., demonstrated good agreement between the extrapolation chamber, films, and Monte Carlo simulation. For previous generation of films, the uncertainty of film measurement was estimated to be ± 10%.^18^ Other studies utilizing MD55‐2 Gafchromic films have reported variations from 13% to 25% in radial dose function, with larger discrepancies at shorter distances.^19^ Additionally, film dosimetry has demonstrated the capability to evaluate the dose perturbation effects caused by stents, calcium deposits, air pockets, and guidewires at distances as close as 0.5 mm.^17^, ^20^, ^21^ EBT‐4 Film, which is the film in use, shows reliable measured doses for accurate dosimetry in various modalities of radiotherapy, including brachytherapy.^22^

The advent of 3D printing presents a novel approach to dose verification in IVBT by allowing the fabrication of 3D‐printed phantoms. This additive manufacturing technique has revolutionized biomedical applications by enabling the fabrication of highly precise models. This process involves digitally slicing a design into 2D layers, which are sequentially printed to construct a 3D object. Such precision allows replication of complex anatomical structures, facilitating realistic dose verification.^23^ Previous studies have demonstrated the utility of 3D‐printed templates in interstitial brachytherapy with improved repeatability and dosimetric accuracy.^23^, ^24^ Additionally, 3D‐printed phantoms have been employed in quality assurance (QA) to validate the dose distribution more accurately than traditional tools, which often fail to replicate the realistic treatment conditions.^25^, ^26^

This study aims to evaluate the dose distribution of intravascular brachytherapy with Sr‐90/Y‐90 sources using 3D‐printed phantoms combined with film dosimetry. Additionally, it investigates the dose perturbation effect caused by the presence of a stent.

## 2 METHODS

### 2.1 Phantom design

The Beta‐Cath 3.5F Delivery System is a hydraulic, catheter‐based device used in IVBT to deliver beta radiation to the coronary arteries while minimizing exposure during transport and storage. The system is connected to a catheter through which the source train—composed of individual, sealed radioactive sources with an inactive radiopaque marker at the end—is hydraulically advanced to the treatment site (Figure 1).^27^

**FIGURE 2:**
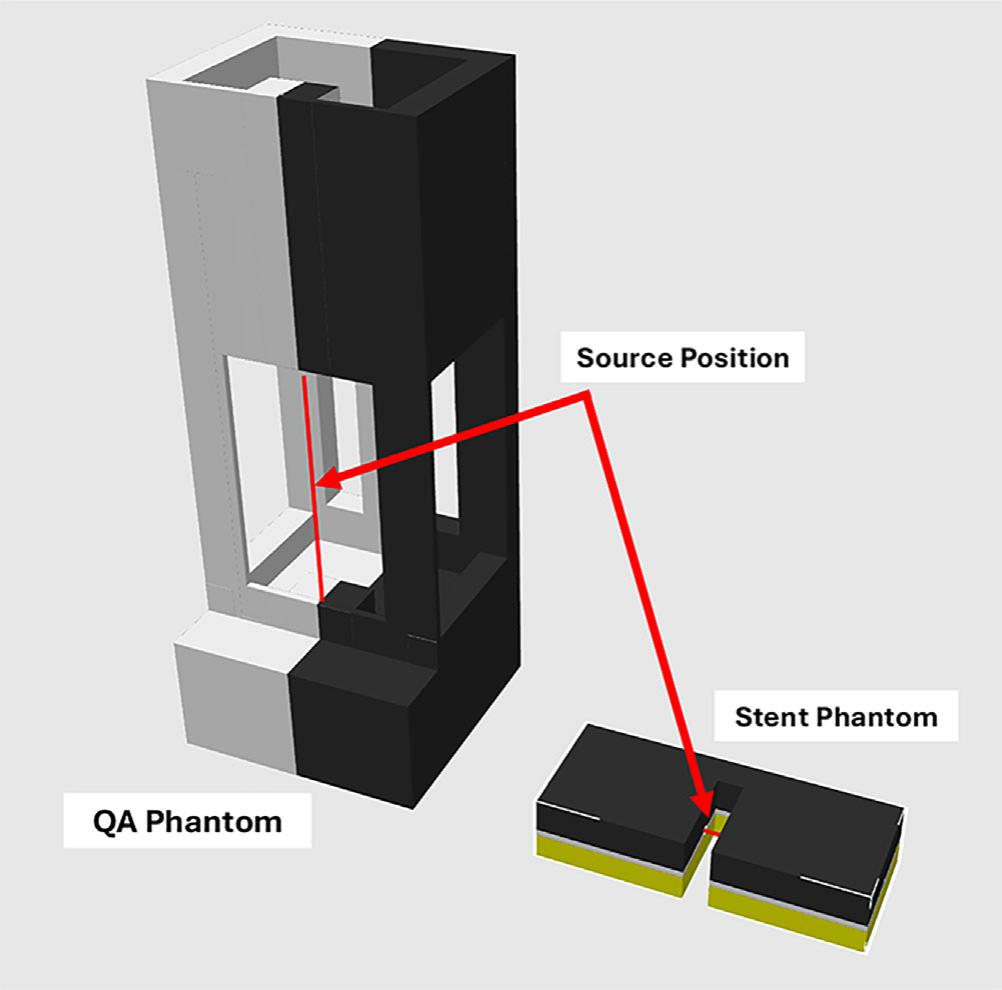
Computer‐aided design (CAD) models of the QA phantom (left) and the stent phantom (right), shown to scale.

Two types of phantoms (Figure 2) were designed and fabricated with 3D printing: (1) Sandwich QA phantom (for general dose verification) and (2) Stent phantom (for evaluating dose perturbation effects due to stents). Reproducibility was tested through multiple trials and by two investigators.

**FIGURE 3:**
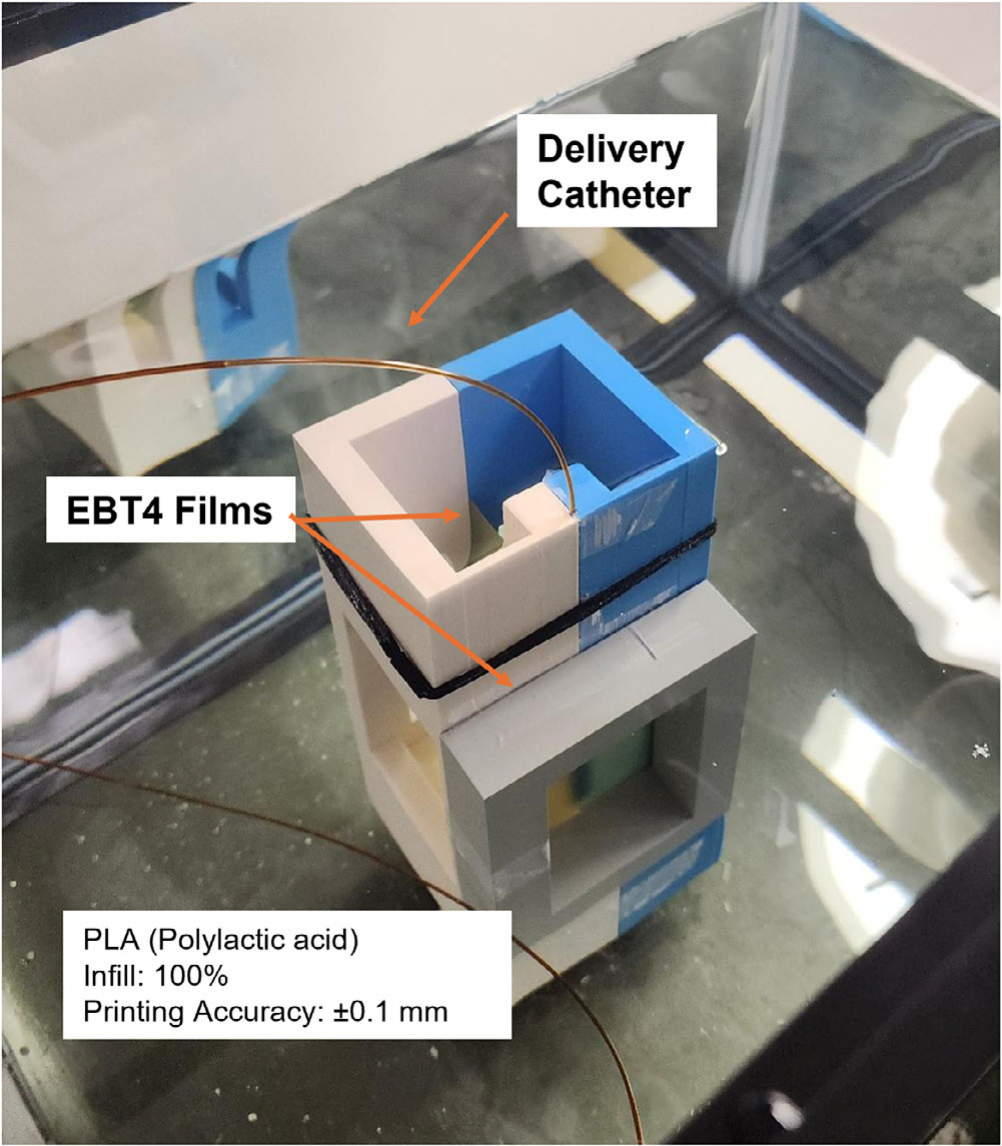
QA phantom setup in water, illustrating the positioning of the radiochromic film and the catheter.

The sandwich QA phantom consists of two interlocking halves that securely enclose both the catheter and film, which ensures precise alignment and reproducibility. The phantom's base dimensions (82.0 × 82.0 × 40.0 mm) provide stability when submerged in water. The central slit, designed to snugly hold the 1.17 mm Beta‐Cath catheter, ensures proper positioning without tilting. A colored rectangular marker was embedded in the phantom to verify that the active layer of the EBT4 film is precisely positioned 2 mm from the catheter center (Figure 3). The phantom's hollow trunk minimizes interactions with PLA material in the measurement region, and an extended neck stabilizes the catheter in a vertical position.

**FIGURE 4:**
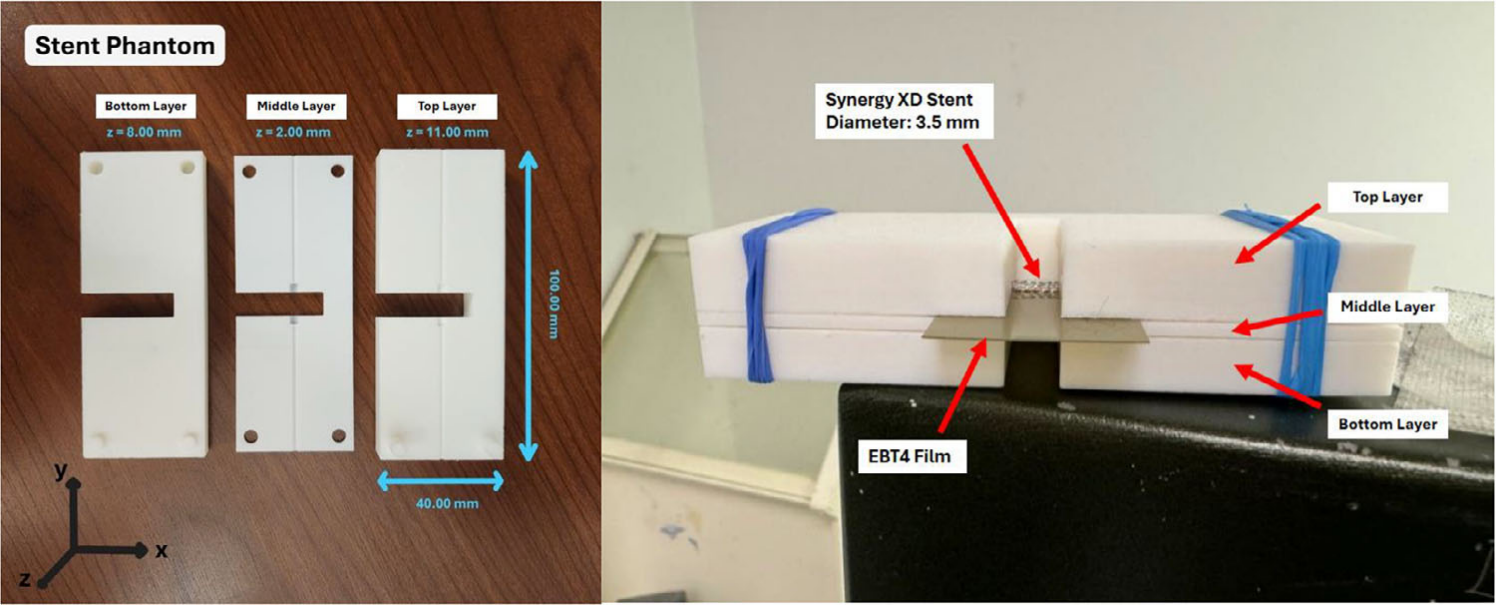
The three‐layer stent phantom shown dismantled (left) and fully assembled (right), with the radiochromic film, stent, and source in place. The phantom consists of three interlocking components that form a capital pi (Π) geometry when assembled.

The stent phantom consists of three interlocking layers, forming a capital pi (Π) shape (Figure 4). The top and middle layers securely hold the catheter and stent in a concentric configuration, ensuring proper alignment. The film is then positioned in direct contact with the stent and lies within the longitudinal plane that passes through the stent's central axis. Additionally, another film can be placed between the middle and bottom layers, allowing for profile measurements at 2 mm from the source center.

**FIGURE 5:**
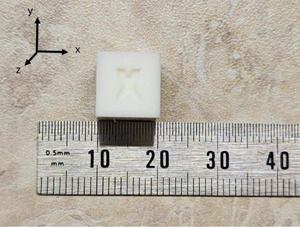
Measurement of a 10 × 10 × 10 mm calibration cube printed together with the custom phantoms, used to verify dimensional accuracy and printer fidelity.

Both phantoms were designed using Autodesk Fusion (Autodesk Inc., San Francisco, California, U.S.) and printed with a Bambu X1 carbon 3D printer (Bambu Lab, Shenzhen, China) with 100% Polylactic Acid (PLA). Figure 2 shows the CAD of both figures to scale. A 10 × 10 × 10 mm calibration cube was printed alongside the phantoms to assess printing accuracy (Figure 5).

### 2.2 Film dosimetry and calibration

To calibrate film response over the dose range relevant to the IVBT treatment, a set of twelve EBT‐4 films (30.0 by 30.0 mm each) was prepared, with doses ranging from 0 to 1200 cGy. Each film was labeled according to its planned dose: 0 cGy, 50 cGy, 100 cGy, 200 cGy, 300 cGy, 400 cGy, 500 cGy, 600 cGy, 700 cGy, 800 cGy, 1000 cGy, and 1200 cGy. The calibration dose was capped at 1200 cGy, consistent with the manufacturer's recommended optimal range of 20 cGy to 1000 cGy for EBT‐4 film. Our maximum measured dose was approximately 7.5 Gy, well within this range. Exceeding this range would increase measurement uncertainty, potentially compromising the reliability of the data.

Irradiation was performed using a 6 MV photon beam on a TrueBeam linear accelerator following recommendations by TG‐235 and justification from previous studies for a beta source. Films should be irradiated with energies that closely match the energy of the source in question. Thus, a 6MV photon beam would be suitable for the Sr‐90/Y‐90 source with energies up to 2.27 MeV.^17^, ^18^, ^28^, ^29^ Films were positioned at a 100 cm source‐to‐axis distance (SAD) with 50 mm of solid water buildup and 20 × 20 cm field size. Following irradiation, films were stored in a controlled environment and scanned 24 h post‐exposure to allow for optical density stabilization.^17^ Scanning was conducted on an Epson 10000XL scanner at 300 dpi, 48‐bit, without color correction. To ensure consistency, films were oriented according to pre‐labeled reference marks when cut.

Calibration curves were generated using the RIT v6.8.64 film dosimetry package, which uses a piecewise interpolation function to translate pixel values into absolute dose values (cGy). The pixel values were extracted from the red channel. The same film scanning procedures were applied to films used during phantom measurements.

### 2.3 Monte Carlo simulation

The Monte Carlo (MC) code used for simulating the phantom and film was GATE, a GEANT4 based simulation tool developed by the international OpenGATE collaboration. The GATE package was downloaded and installed on a Linux‐based workstation running Ubuntu, where different photon and particle interactions were simulated, including Compton, photoelectric, and pair production for photon particles with electron ionization, the Continuous Slowing Down Approximation (CSDA) model, and Bremsstrahlung for electrons and positrons.

The geometry of the Sr‐90/Y‐90 train source was first constructed in the system. The strontium body is a sealed source with a size of 2.5 mm x 0.38 mm. The train jacket is stainless steel 304 (an alloy of iron, carbon, chromium, and nickel) with an inner diameter of 0.42 mm and an outer diameter of 0.47 mm. The source train has a 40 mm active length, consisting of 16 radioactive sources and two radiopaque markers. The Pt/Ir end markers were not modeled. The delivery catheter was modeled as a polyethylene tube.

The MC code, initially developed for Y‐90 disc source dose distribution simulations, was modified for Sr‐90/Y‐90 source train simulations. Validation was performed by comparing the simulated dose distribution to the vendor‐provided reference data.^29^, ^30^ The thickness of the steel jacket was simulated. The sum of squared differences in PDD between 0.75 mm and 6.5 mm, evaluated at 0.25 mm intervals, was calculated relative to the vender's data. The thickness that most closely matched was 0.025 mm wall thickness of the jacket, with a sum of square differences of 0.076. The average of the absolute value of percent differences was 2.87%, with the largest percent difference being 10.1% at a depth of 4.0 mm compared to the vender's data. The simulations were conducted in water, and the PDD curve was calculated by simulating a film perpendicular to the source.

**FIGURE 6:**
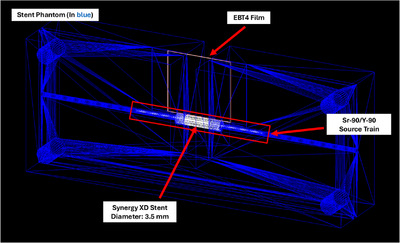
Monte Carlo simulation model of the stent phantom with the radiochromic film positioned at the central plane.

The phantom STL files were directly imported into the simulation codes, with the film placed at the measurement location (Figure 6). For the sandwich QA phantom, the Sr‐90/Y‐90 source train was suspended in water, with a thin, rectangular detector positioned perpendicular to the source for PDD. For the stent phantom, the stent was modeled as a platinum‐chromium (PtCr) alloy mesh, consisting of platinum, chromium, iron, nickel, and molybdenum. The simulated stent had a length of 16 mm, a strut thickness of 0.0031 inches (0.079 mm), and a diameter of 3.0 mm. The simulation was executed with 2 × 10⁷ primaries to ensure an uncertainty of less than 2%.

### 2.4 Experimental setup and irradiation procedure

For dose measurements, two phantoms—the sandwich QA phantom and the stent phantom—were carefully assembled. The delivery catheter and films were positioned in their designated slots. For the sandwich QA phantom, the PDD film's alignment relative to the catheter surface was verified by a second observer to maintain consistency. Once alignment was confirmed, the two halves of the phantom were securely joined, and the assembled phantom was submerged in the water tank (Figure 3). For the stent phantom, the catheter was marked to align the center of the 40 mm source with the stent. The films were positioned in direct contact with the stent and lie within the longitudinal plane that passes through the stent's central axis (Figure 4). The niche to place the PDD film was predetermined based on the diameter of the stent and film thickness. Films were kept at the same position with and without the stent for directly evaluating the dose impact from the stent. Once alignment was verified, the three phantom layers were securely joined, and the assembled phantom was submerged in the water tank.

The catheter was loaded with the Sr‐90/Y‐90 source train using the Novoste Beta‐Cath system. Before introducing the source, the Beta‐Cath system was inspected for any fluid leakage. With the fluid bag attached, the system gate closed, and the source positioned in the return location, a syringe was used to inject 2 mL of sterile water to test for leakage. If fluid returned to the bag, the system was deemed safe and leak‐free. Before beginning film irradiation, a camera was placed to monitor the source location and measure the irradiation time. Once the recording view was verified, the gate was then opened and set to the “send” position, allowing fluid pressure to drive the source to the designated dwell position within the phantom. The source remained in this position for approximately 2 min to ensure sufficient exposure, with the exact exposure time determined from the video recording. The highest dose measured was around 7 Gy to have shorter experimental sessions and minimize exposure to operators. After exposure, the source was returned to the storage position, and the gate was closed.

Following the measurement, the phantom was disassembled, and the films were retrieved and labeled with the experiment date, time, and trial number for identification. The films were then processed using the same scanning and dosimetry protocol as in the film calibration. PDD curves were exported as ASCII files from RIT v6.8.64 software and analyzed using OriginPro 2025a.

### 2.5 Percent depth dose analysis

**FIGURE 7:**
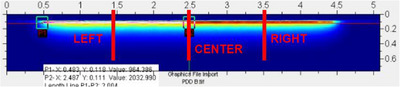
Image of the PDD film measurements showing the dose distribution heatmap. PPD data are extracted along the red line and labeled as LEFT periphery, CENTER, and RIGHT periphery.

The PDD ASCII file for the sandwich QA phantom was extracted from RIT v6.8.64 for three measurement locations, left peripheral, center, and right peripheral, as shown in Figure 7, and imported to OriginPro 2025a for analysis. For the Stent Phantom, the PDD data was collected solely from the center and processed in the same manner.

To ensure data accuracy, points with little to no detected dose were excluded. The initial readings, recorded in the units of cGy vs. cm, were converted to Gy/sec vs. mm. To minimize errors from source sag, the average of the three measured PDD curves were taken. The resulting curve was fitted using the third‐order exponential polynomial equation used for a planar Y‐90/Sr‐90 source, as described in Soares et al.^18^, ^31^:

$$\;{\left[D\left(z,r_{0}\right)/D\left(z_{0},r_{0}\right)\right]}_{s}=\exp\left(a_{s}+b_{s}z+c_{s}z^{2}+d_{s}z^{3}\right)$$

PDD curves obtained from the stent phantom, both with and without the stent, were analyzed and compared to the Monte Carlo simulation and vendor reference data.

## 3 RESULTS

### 3.1 Film calibration

The calibration curve, generated by using the piecewise polynomial function, preserves the shape of the data in comparison to linear and cubic spine functions.

### 3.2 Phantom printing accuracy

The calibration cube for verifying printing accuracy and the phantom components were printed using the printing parameters listed in Table 1. The cube exhibited a max printing uncertainty of 0.09 mm in the *x* and *z* direction (Figure 5). Based on this evaluation, the depth in the x and z direction of the phantom was overestimated by 0.10 mm.

**TABLE 1:** Summary of 3D printing parameters used for phantom fabrication.

Printing parameters	
Filament diameter (mm)	1.75 mm
Nozzle diameter (mm)	0.4 mm
Extruder temperature (°C)	220°C
Table temperature (°C)	55°C
Layer thickness (mm)	0.08 mm
Maximum print speed (mm/s)	450 mm/s
Minimum print speed (mm/s)	20 mm/s
Fill Structure	Rectilinear
Infill Density (%)	100%
Material	PLA

### 3.3 Depth dose distribution

#### 3.3.1 QA phantom

**FIGURE 8:**
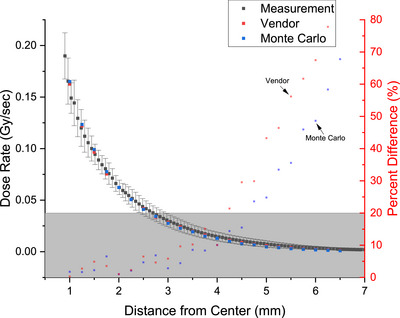
Fitted parameters for the QA phantom based on third‐order exponential polynomial function.

The resulting parameters from the third‐order exponential polynomial curve fitting of the measured PDD data are displayed in Figure 8. The fitting process yielded an r‐squared value of 0.998. The reduced chi‐square value of 2.53 × 10^−6^ further demonstrates the model's strong goodness‐of‐fit. The residuals analysis revealed minimal scatter around zero, with no discernible systematic deviations. The error bars at each point denote the standard deviation across six different measurements performed on six different days by two different investigators.

The absolute dose comparison was performed to evaluate the accuracy of the QA phantom against vendor data and Monte Carlo simulations. At a clinically relevant distance of 2 mm from the vessel center, the local percent difference between the QA phantom measurements and the Monte Carlo simulation was 1.012%, based on the institution's source calibration as of 2/2/2024.

Across the clinically significant range of 2 to 5 mm from the vessel center (corresponding to a maximum vessel diameter of 8 mm), the local percent difference remained consistently below 20%. The comparison of the absolute dose values is presented in Figure 8, highlighting the close agreement between the QA phantom, Monte Carlo simulations, and vendor‐provided data across multiple radial distances. The mean percentage difference for all measurements from 1.00 mm to 6.50 mm was 6.0%, with a standard deviation of 4.4%. Confidence interval of 3.32% and 8.52% further confirm that the observed errors are within clinically acceptable limits and fall within this range 95% of the time.

To further quantify the measurement accuracy, root mean squared error (RMSE) and mean absolute error (MAE) were calculated for comparisons with both references. The RMSE was 1.96 × 10^−3^ and 2.32 × 10^−3^ and the MAE was 1.77 × 10^−3^ and 2.15 × 10^−3^ when compared with the vendor and Monte Carlo data, respectively.

#### 3.3.2 Stent phantom

The dose distribution for the Stent Phantom, measured both with and without the stent, was analyzed using the third‐order exponential polynomial function to model the percent depth dose.

**FIGURE 9:**
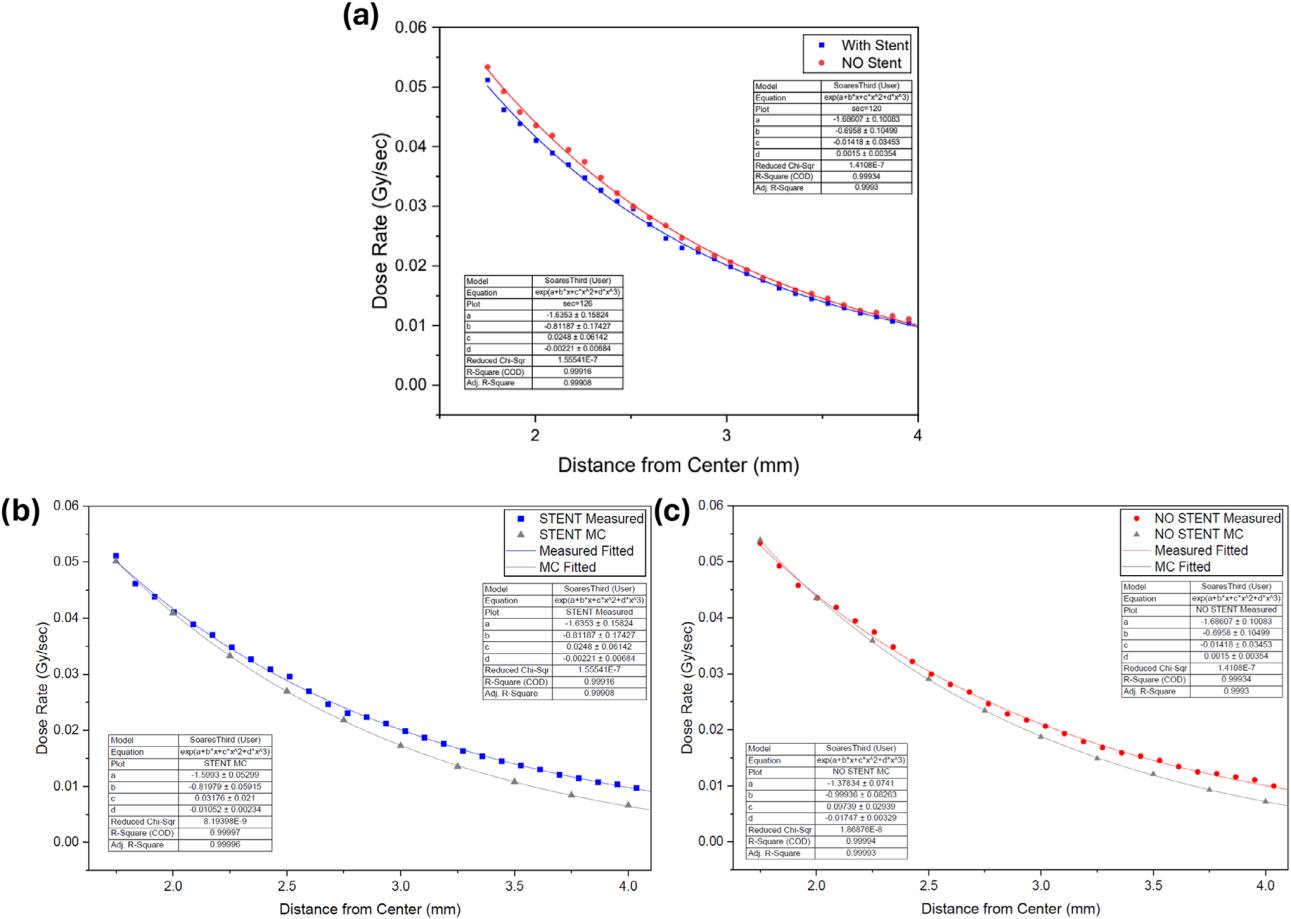
(a) Comparison of measured PDD curves from the stent phantom with and without the stent, along with their respective curve fits. (b) Comparison of the measured PDD with the stent in place versus the Monte Carlo simulation. (c) Comparison of the measured PDD without the stent versus the Monte Carlo simulation.

For the no‐stent configuration, the fitting yielded an R‐squared value of 0.999, indicating an excellent correlation between the measured data and the modeled dose distribution. The reduced chi‐square value was 1.41 × 10^−7^, which is close to zero, further confirming the quality of the curve fitting and suggesting that the model closely matches the measurement data (Figure 9).

Similarly, for the with‐stent configuration, the R‐squared value was 0.999, again reflecting a nearly perfect fit. The reduced chi‐square value was 1.56 × 10^−7^, indicating strong agreement between the model and the measurement, even with the added complexity introduced by the stent structure.

To evaluate the agreement between the measured percentage depth dose values and the Monte Carlo simulations, the RMSE and MAE were calculated for the No‐Stent and With‐Stent datasets. For the No‐Stent dataset, the RMSE was 3.57 × 10^−2^ and the MAE was 3.27 × 10^−2^ For the With‐Stent dataset, the RMSE was 3.84 × 10^−2^ and the MAE was 3.55 × 10^−2^. There was a slightly higher difference in the With‐Stent dataset, suggesting that the presence of the stent may increase dose discrepancies between the expected and delivered.

To statistically assess the impact of the stent on the dose distribution, a paired *t*‐test was performed to compare the PDD values measured with and without the stent. The results of the *t*‐test showed a *t*‐statistic of −6.591 with 36 degrees of freedom. The corresponding *p*‐value was < 0.0001, indicating a highly significant difference between the two configurations. This confirms that the stent has a statistically significant impact on the PDD values.

Additionally, the average percent difference in PDD values between the with‐stent and no‐stent measurements within the clinically relevant depth of 2–5 mm was calculated to be 4.26%.

To further quantify the stent`s perturbation effect, the dose reduction factor (DRF) was also calculated using the formula:

$$\text{DRF}=1-\frac{\text{dose}\;\text{with}\;\text{stent}}{\text{dose without stent}}$$

The DRF values ranged from a minimum of 1.18% at a depth of 2.51 mm to a maximum of 7.92% at a depth of 3.87 mm, with an average DRF of 4.5%. These values indicate that the stent attenuates the delivered dose by approximately 4.5% on average, with variations depending on the depth of interest. In the region of clinical interest (2–5 mm), the DRF showed consistent attenuation trends.

## 4 DISCUSSION

This study explored the feasibility of using 3D‐printed phantoms to verify the dose distribution of Sr‐90/Y‐90 sources in intravascular brachytherapy, focusing on two primary designs: a QA phantom and a Stent‐specific phantom. The results demonstrated that 3D‐printed phantoms, fabricated from a single material, can replicate complex 3D geometries, ensuring accurate positioning for film dosimetry, and providing reproducible dose measurements in high‐gradient environments.

The use of 3D‐printed phantoms addressed many of the limitations inherent in conventional dosimetry techniques, particularly for IVBT. By incorporating material heterogeneities, such as the platinum‐chromium (PtCr) alloy used in stents, the phantoms enabled more accurate simulations of real‐world clinical scenarios. Furthermore, the modular design of the 3D‐printed phantoms allows for precise and reproducible alignment, mitigating the positional error. In particular, the QA phantom was engineered to securely hold the Beta‐Cath catheter, ensuring consistent film placement from the source axis, which was critical for accurate measurement of PDDs. The stent phantom facilitated dose verification in the presence of a stent by stabilizing it within a three‐layered structure, reproducing realistic treatment geometries where metallic components can perturb dose distributions. The findings highlight the potential of advanced 3D‐printed phantoms to significantly enhance the accuracy and clinical relevance of dose verification in IVBT.

The third‐order exponential polynomial curve fitting provided an excellent fit for the measured PDD data, with an R‐squared value of 0.998 and a reduced chi‐square value of 2.53 × 10^−6^ for the QA Phantom. The fitting equation was chosen due to its proven accuracy in representing the dose distribution for intravascular brachytherapy sources.

When comparing the QA phantom measurements to Monte Carlo simulations, the local percent difference at 2 mm from the vessel center was 1.012%, which is within clinically acceptable limits. The consistency of the local percent difference across the 2 to 5 mm range reinforces the QA phantom's ability to accurately replicate clinical conditions. The overall mean percentage difference of 6.0% with a standard deviation of 4.4%, and the 95% confidence interval (3.32% to 8.52%), indicates that the phantom's accuracy is reliable and falls well within tolerance thresholds. RMSE and MAE values for the comparison with both vendor data and Monte Carlo simulations are very low, further supporting the precision of the QA phantom. These results confirm that the phantom provides accurate dose measurements, with very minimal variation when compared to the reference data. The measured data showed close agreement with Monte Carlo simulations and vendor references, with discrepancies as low as 1.012%.

In the analysis of the Stent Phantom, the R‐squared values of 0.999 for the No‐Stent configuration and 0.999 for the With‐Stent configuration indicate nearly perfect fits for both measurement setups. However, the presence of the stent introduces slight discrepancies, as seen in the slightly higher RMSE and MAE values for the With‐Stent measurements, compared to the No‐Stent measurements. This suggests that the stent introduces some variation in dose delivery, which could impact treatment accuracy.

The inclusion of a stent‐specific phantom in this study provided important insights into dose perturbations caused by metallic stents during IVBT. The analysis showed a statistically significant difference in PDD between stent and non‐stent configurations (*p* < 0.0001), with an average DRF of 4.5% within the clinically relevant range. Assuming 4.3% dose reduction at the prescription depth of 18.4 and 23 Gy would result in an actual delivered dose of 17.6 Gy and 22.0 Gy, respectively. Previous studies show that the success of IVBT is dose dependent. The rate of restenosis for the group treated with 12 Gy, 15 Gy, and 18 Gy was 21%, 16%, and 15%, respectively.^32^ While the dose reduction of 4.3% from the stent may result in a minimal change in the rate of restenosis, with factors such as plaque buildup and heart motion, dose reduction may increase to up to 30%.^33^ These findings suggest that localized underdosing may occur near stent struts compromising treatment efficacy and contributing to restenosis within the treatment site.^34^ Users should understand the dosimetric impact of the stent before utilizing the Beta‐Cath system with patients with previous stent treatments.

Contrary to the conventional expectation that metallic components increase local dose deposition via enhanced scattering, the results of this study indicate a different trend. Beta particles typically have a range of about ten centimeters in air, but a few millimeters in tissue.^4^ Due to factors such as the stent geometry, material composition, and thickness, dose perturbation may have occurred. A previous Monte Carlo study investigated different metallic compositions and radius of different stents. The study showed a dose reduction of 5% to 20% for beta sources, consistent with this study measurements. The consistent attenuation across 2‐ to 4‐mm depth is observed, supporting the conclusion that metallic stents have a measurable impact on local dose delivery and should be accounted for during treatment planning in IVBT. Accurate modeling and verification of dose distributions in the presence of stents are therefore essential to ensure optimal treatment outcomes.

EBT‐4 film is well‐suited for capturing the steep dose gradients characteristic of Sr‐90/Y‐90 beta radiation. The high spatial resolution of the film enabled detailed mapping of dose distributions in the 0–10 Gy range. The measurement time was limited to around 2 min, corresponding to a delivered dose of approximately 7.5 Gy. This approach facilitated simple and reproducible procedures, improved the statistical reliability of the data, minimized radiation exposure to investigators, and ensured safe working conditions. Although the 10 Gy range does not encompass the typical prescription dose for IVBT treatment (18.4–23 Gy, depending on vessel length and diameter), this was not a concern for the present study. The primary objective was to evaluate the relative dose perturbation caused by the presence of coronary stents during IVBT, rather than to reproduce the full clinical prescription dose. Dose perturbation was expressed as the percentage difference between measurements with and without stents. Because this metric is relative, it is independent of the absolute dose and can be readily scaled to clinical prescription levels. If high‐dose range is involved, such as a prolonged exposure time, where doses are greater than 10 Gy, EBT‐XD with a green channel scanning would be recommended for improved dosimetric accuracy.^17^

Several other dosimeters are also reported for dose measurements in IVBT and brachytherapy in general. Thermoluminescent Dosimeters (TLDs) have been used to measure absolute dose rates near beta sources.^35^, ^36^, ^37^ These detectors offer high point‐dose accuracy but lack the spatial resolution needed to resolve steep dose gradients. Furthermore, they are typically limited to doses below ∼30 Gy per fraction.^38^, ^39^ Ionization chamber arrays, particularly liquid‐filled designs, have been applied to HDR brachytherapy.^40^ These arrays provide high sensitivity and accuracy but are sensitive to positional errors and exhibit variability based on the individual chamber's characteristics, making them less ideal for IVBT's small‐field, high‐gradient settings.^41^ Polymer gel dosimeters allow 3D volumetric dose verification and are useful in HDR contexts. Upon irradiation, the polymerization of the gel correlates with absorbed dose, which can be imaged via MRI or optical scanning. However, gel dosimetry is labor‐intensive, sensitive to environmental conditions, and less accurate in regions of high dose gradients near the source.^42^

Despite the promising results, this study has several limitations. First, the 3D‐printed phantoms were fabricated using PLA with a physical density of 1.24 g/cc, which does not fully mimic the heterogeneity of human tissues.^43^ It was shown that the standard PLA exhibited a mean Hounsfield Unit (HU) value of –68.58 compared to 34.59 for the commercial bolus. In absolute dose measurements using a 10 × 10 cm^2^ field, the charge collected with standard PLA bolus was 0.009 nC higher than that measured with solid water. Additionally, they showed concordance correlation coefficients to assess agreements of commercial bolus compared to solid water and standard PLA. The correlation coefficients are 0.9997 for solid water and 0.9987 for standard PLA.^44^ In our study, the concern of PLA affecting dose measurements is minimal, as no PLA material is in direct contact with the radiation source. However, minor effects due to side scatter from surrounding PLA structures cannot be entirely ruled out. Further studies are needed to explore alternative 3D printing materials that may better simulate tissue properties, such as soft tissue or vessel walls. Second, the resolution of 3D printing and manual film alignment may introduce potential sources of errors, particularly in the stent phantom, where precise positioning of the film around the stent struts is critical. An automated position verification mechanism should be incorporated in the future design to minimize these types of errors.

In addition to the composition and geometry of the 3D‐printed model itself, the variations in stent composition and geometry can significantly influence the dosimetric impact. Changes in the stent's diameter or shape may alter scattering conditions and attenuation characteristics. In this study, the diameter of the stent was kept to 3 mm with no variation during measurement which does not fully reflect the variability during treatment. A previous study investigates stent expansion and thickness effects using the Monte Carlo simulation where regardless of the thickness or expansion, dose reduction was observed up to 20%.^45^ Another Monte Carlo–based manuscript is currently in preparation, focusing on dose delivery variations resulting from differences in plaque building, vessel diameter, stent thickness, and heart motion.^46^ Expanding the current design to incorporate dynamic conditions, such as catheter movement, blood flow, or vessel curvature, would provide a more comprehensive evaluation of IVBT dose delivery. Additionally, 3D‐printed phantoms could be adapted for use in other brachytherapy applications, such as prostate or eye plaque treatments, where precision dosimetry is equally critical. By integrating 3D‐printed phantoms with emerging technologies like real‐time dosimetry or 3D‐printed tissue‐equivalent stents, this approach has the potential to transform quality assurance processes across a variety of brachytherapy contexts.

## 5 CONCLUSION

Overall, the comparison among film dosimetry measurements, Monte Carlo simulations, and vendor‐provided dose data demonstrated strong agreement within clinically acceptable limits. The presence of metallic stents introduced slightly greater variation as reflected by an average dose reduction factor of 4.5% and statistically significant dose perturbations. These results indicate that replicating similar measurement setups in clinical environments could anticipate dose deviations generally within 3 to 9%, contingent on phantom configuration and the presence of dose‐perturbing materials such as stents.

This study demonstrated that 3D‐printed phantoms are a feasible and effective tool for dose verification in intravascular brachytherapy. Film‐based dosimetry successfully provided high‐resolution dose measurements, particularly PDDs. The experimental setup ensured accurate positioning of the source, maintaining a centered and stable orientation to avoid sagging. These findings validate the use of film dosimetry within 3D‐printed phantoms as a reliable quality assurance method for IVBT.

The study's inclusion of a stent‐specific phantom highlights the importance of considering stent‐induced dose perturbations during IVBT. By providing a straightforward workflow to evaluate dose distributions in the presence of stents, this approach can serve as a valuable investigation tool for analyzing new stent designs. The workflow is easy to implement, allows for relative dose comparisons, and closely replicates clinical conditions, ensuring practical applicability in treatment planning and QA. These tools can be used to identify and mitigate dose variations caused by stents and other materials such as plaque to improve the precision and safety of IVBT procedures.

## AUTHOR CONTRIBUTIONS

Lyu Huang and Jessica Jung led the project, conducted the research and analysis, and drafted the manuscript. Yijian Cao conceptualized the study and developed the core methodologies. Nicholas Coupera contributed specifically to the development and implementation of the Monte Carlo simulation. Lyu Huang and Jenghwa Chang provided supervision, mentorship, and critical revisions to the manuscript.

## CONFLICT OF INTEREST STATEMENT

The authors declare no conflicts of interest.
